# Mobile Telephone Use and Reaction Time in Drivers With Glaucoma

**DOI:** 10.1001/jamanetworkopen.2019.2169

**Published:** 2019-04-12

**Authors:** Nara G. Ogata, Fábio B. Daga, Alessandro A. Jammal, Erwin R. Boer, Linda L. Hill, James M. Stringham, Remo Susanna, Felipe A. Medeiros

**Affiliations:** 1 Visual Performance Laboratory, Duke Eye Center, Department of Ophthalmology, Duke University, Durham, North Carolina; 2 Department of Ophthalmology, University of São Paulo, São Paulo, Brazil; 3 Entropy Control Inc, La Jolla, California; 4 Division of Preventive Medicine, Department of Family Medicine and Public Health, University of California, San Diego, La Jolla

## Abstract

**Importance:**

Combining mobile telephone use with driving is not unusual. However, distracted driving limits driving performance because of limited capacity for persons to divide attention.

**Objectives:**

To investigate the frequency of mobile telephone use while driving and to assess whether patients with glaucoma had a disproportionate decrease in driving performance while conversing on a mobile telephone compared with healthy participants.

**Design, Setting, and Participants:**

Cross-sectional study of surveys collected from 112 patients with glaucoma and 70 control participants investigating mobile telephone use while driving. A randomly selected subgroup of 37 patients with glaucoma and 28 controls drove in a driving simulator to investigate peripheral event detection performance during distracted driving at the Visual Performance Laboratory, Duke University, Durham, North Carolina. Data collection was performed from December 1, 2016, through April 30, 2017.

**Exposures:**

Participants answered a survey and submitted to a driving simulation test with and without mobile telephone use.

**Main Outcomes and Measures:**

Survey answers were collected, and distracted driving performance, assessed by reaction time to peripheral stimuli, was analyzed.

**Results:**

Of the 182 participants who answered the survey, the 112 participants with glaucoma included 56 women (50.0%) and had a mean (SD) age of 73.6 (9.6) years. The 70 controls included 49 women (70.0%) and had a mean (SD) age of 68.4 (10.9) years. When asked about mobile telephone use while driving, 30 patients with glaucoma (26.8%) admitted rarely using and 2 (1.8%) sometimes using it. In the control group, 20 participants (28.6%) admitted rarely using and 2 (2.9%) sometimes using the telephone while driving (*P* = .80). Reaction times to peripheral stimuli were significantly longer among patients with glaucoma compared with controls during mobile telephone use (median [interquartile range], 1.86 [1.42-2.29] seconds vs 1.14 [0.98-1.59] seconds; *P* = .02). Compared with driving performance while not using a mobile telephone, the mean (SD) increase of 0.85 (0.60) second in reaction time while conversing on the mobile telephone among patients with glaucoma was significantly greater than the mean (SD) increase of 0.68 (0.83) second for controls (*P* = .03).

**Conclusions and Relevance:**

This study’s findings indicate that patients with glaucoma use mobile telephones while driving as frequently as healthy participants. However, the findings also suggest that patients with glaucoma may experience a greater decline than healthy participants in their ability to detect peripheral events while driving when also talking on a mobile telephone. Patients with glaucoma should be informed that they may have a higher driving risk that may be worsened by distractions, such as mobile telephone use.

## Key Points

**Question:**

Do drivers with glaucoma show a greater decrease in performance during distracted driving compared with controls?

**Findings:**

In a cross-sectional study of 37 patients with glaucoma and 28 control participants using a driving simulator experiment, reaction times to peripheral stimuli while driving and using a mobile telephone were significantly longer among patients with glaucoma vs controls. Compared with driving without using a mobile telephone, increase in reaction time while conversing on a mobile telephone was significantly greater for patients with glaucoma than for controls.

**Meaning:**

When talking on a mobile telephone while driving, patients with glaucoma may experience a greater decline in their ability to detect peripheral visual events than do persons without glaucoma.

## Teaser

This cross-sectional study compares reaction times to peripheral stimuli while driving and using mobile telephones among patients with glaucoma vs healthy controls.

## Introduction

Driving is the main mode of transportation, and the ability to drive is strongly associated with quality of life.^1,2,3^ The National Safety Council's annual injury and fatality report has estimated that the use of mobile telephones while driving is responsible for 26% of motor vehicle crashes.^4^ Most of these crashes involve drivers who are distracted by talking on mobile telephones.

Distracted driving limits driving performance because of humans’ limited capacity to divide attention.^5^ When driving and talking on mobile telephones, drivers have limited ability to divide attention and may fail to detect hazardous situations. Although the outcomes of distracted driving and divided attention have been well studied in the general population, there is a paucity of work conducted among patients with coexisting morbidities. As a highly visual task, the ability to drive may be significantly compromised in individuals with visual impairment. When faced with the additional challenge imposed by divided attention from mobile telephone use, participants with visual impairment may have even greater compromise in the ability to drive safely compared with healthy participants.

Glaucoma is a progressive optic neuropathy and the leading cause of irreversible visual impairment worldwide.^6^ Glaucoma can affect several aspects of quality of life and impair performance among a broad array of activities, including driving.^7^ Although glaucoma has been previously associated with increased risk for motor vehicle crashes,^5,8^ the conditions associated with increased risk in this population have not been well clarified. Gangeddula and colleagues^9^ have shown that the functional field of view of patients with glaucoma experiences a disproportionate reduction compared with that of healthy individuals under increased cognitive loading. Moreover, when drivers are cognitively distracted, their eyes are fixated on a single target and eye dispersion is reduced.^10^ Because of an already compromised field of view, patients with glaucoma may have a disproportionate decrease in driving performance during mobile telephone conversation compared with controls, placing them at an even greater risk for motor vehicle crashes in the presence of distracted driving.

In this study, we investigated the prevalence of mobile telephone use among drivers with glaucoma. We hypothesized that, since glaucoma can remain asymptomatic until late stages, most drivers with glaucoma would remain unaware of their limitations and not necessarily limit their use of mobile telephones while driving. We also investigated driving performance during mobile telephone use through driving simulation, hypothesizing that drivers with glaucoma would be associated with a greater decrease in performance under distracted driving conditions compared with healthy individuals.

## Methods

For this cross-sectional study, participants were drawn from a prospective study designed to evaluate functional impairment in patients with glaucoma that was conducted at the University of California, San Diego. Data collection was performed from December 1, 2016, through April 30, 2017. Written informed consent was obtained from all participants, and the University of California, San Diego institutional review board approved all methods. Methods adhered to the tenets of the Declaration of Helsinki for research involving humans,^11^ and the study was conducted in accordance with the regulations of the Health Insurance Portability and Accountability Act. This study followed the Strengthening the Reporting of Observational Studies in Epidemiology (STROBE) reporting guideline.

Participants underwent a comprehensive ophthalmologic examination, which included review of medical history; slitlamp biomicroscopy, gonioscopy, dilated ophthalmoscopic examination, and stereoscopic optic disc photography; and measurement of visual acuity, intraocular pressure, and standard automated perimetry (SAP). Patients with coexisting ocular or systemic disease that could affect the optic nerve or the visual field were excluded from the study. We also excluded participants who were not active drivers.

Glaucoma was defined by the presence of 2 or more consecutive abnormal SAP test results, defined as a pattern SD with *P* < .05 and/or glaucoma-hemifield test results outside normal limits. Healthy controls in this study were recruited from the general population, university staff, and employees through advertisements. To be included, controls’ eyes had to meet the following criteria: (1) intraocular pressure below 22 mm Hg and no history of elevated intraocular pressure; (2) normal ophthalmologic examination results; (3) a normal appearance of the optic disc on masked grading of stereophotographs; and (4) at least 2 reliable normal visual fields in both eyes, which were defined as a pattern SD within the 95% CI and a glaucoma-hemifield test result within normal limits.

### Standard Automated Perimetry

All participants underwent SAP testing using the 24-2 Swedish Interactive Thresholding Algorithm Standard strategy of the Humphrey Field Analyzer II (Carl Zeiss Meditec, Inc). Only patients with reliable test results (≤33% fixation losses or false-negatives and ≤15% false-positives) and open angles on gonioscopy were included.

The SAP mean deviation and mean sensitivity were evaluated as factors for assessment of the overall integrity of the visual field, which is indicative of disease severity. The values are provided in decibels, and lower values indicate greater visual field loss. The SAP testing is routinely performed monocularly. Therefore, to gauge binocular visual field loss, sensitivities of the monocular SAP tests of both eyes were combined to calculate an integrated binocular visual field mean sensitivity, according to the binocular summation model described by Nelson-Quigg and colleagues.^12^

### Distracted Driving Survey

Participants were administered a distracted driving survey containing 58 questions, previously developed and validated in other populations^13,14^ but modified to target older adults. The following items from the survey were used in the analyses to focus on estimating the prevalence of distracted driving:

1. On a typical day that you drive, approximately how much time do you spend driving? The possible responses were fewer than 30 minutes, 30 to 60 minutes, 1 to 2 hours, and more than 2 hours.
2. How many miles do you drive in a typical week? Possible responses were from 1 to 10 miles, 11 to 30 miles, 31 to 50 miles, 51 to 100 miles, and more than 100 miles.
3. Of the amount of time that you spend driving on an average day, how much time do you spend talking on a handheld mobile telephone? Possible responses were never (only emergency or 911 calls), rarely (<10% of the time), sometimes (25% of the time), often (50% of the time), and frequently (>75% of the time).
4. Even if you do not use a mobile telephone when driving, are you capable of driving safely if talking on a handheld mobile telephone? Possible responses ranged from 1 (not capable at all) to 5 (very capable).

### Driving Simulation

A random subgroup (50% of participants) responding to the survey was invited to undergo a protocol to investigate the association of mobile telephone use while driving with changes in driving performance. A high-fidelity driving simulator (Realtime Technologies Inc)^1^ allowed an assessment of driving performance during situations that would be difficult to recreate in the real world without imposing significant risks to the participants.

The protocol consisted of an assessment of the ability to divide attention while driving on a winding country road for 1 minute 30 seconds. The vehicle speed was automatically kept constant at 45 mph (to convert to kilometers per hour, multiply by 1.6), so that the driver only had to operate the steering wheel. Peripheral diamond-shaped targets were presented at approximately 20° of visual angle in the upper right and upper left of the driving simulator. These peripheral stimuli changed shape randomly throughout the test to a camera symbol (eFigure in the Supplement), and participants were instructed to press a button on the steering wheel when they detected the change. The ability to divide attention was assessed by measuring the reaction times to press the button when the symbol changed.^15^ Between 7 and 9 stimuli were presented. Each stimulus had a duration of 1 second, and the driver had 6 seconds to answer to each stimulus. If the driver missed the symbol, the maximum time for pressing the button (6 seconds) was considered the reaction time. Previous studies also used reaction times to assess the ability to divide attention in driving experiments.^5,8,16,17,18^

Before the experiment, each participant performed a practice drive to become familiar with the simulator. Then, participants received instructions and practiced the telephone task with the experimenter, using the mobile telephone.

Participants were required to complete the divided-attention task first without the mobile telephone and then with the mobile telephone. When using the mobile telephone, participants had to perform a previously validated mobile telephone task based on the modified grammatical reasoning (working memory) task.^19,20,21^ This task consisted of hearing a recorded 5-word sentence every 10 seconds through the mobile telephone. After each sentence, the driver was asked if the sentence made sense. Seven seconds after the sentence began, the driver was asked, “Last word?” and was given an additional 3 seconds to answer. The host engaged in the telephone task while seated outside the driving simulator room and hence could neither observe the participant while driving nor receive any clues regarding route progress. The mobile telephone task was intended to replicate a very casual mobile telephone conversation that does not require any mental rehearsal or recall intervals of greater than 3 seconds.^22^

### Statistical Analysis

Univariable and multivariable regression models were applied to investigate differences in reaction times between controls and participants with glaucoma for driving simulation with and without mobile telephone use, and also to investigate the association of severity of visual field loss on reaction times. Generalized estimating equations^23^ were used to take into account multiple correlated measurements for each participant. All statistical analyses were performed using Stata, version 14 (StataCorp). The 2-sided α level (type I error) was set at .05.

## Results

Of the total 182 participants who answered the survey, the 112 participants with glaucoma included 56 women (50.0%) and had a mean (SD) age of 73.6 (9.6) years, and the 70 controls included 49 women (70.0%) and had a mean (SD) age of 68.4 (10.9) years. Table 1 summarizes demographic and clinical characteristics of individuals who responded to the survey. There was no statistically significant difference between groups regarding their answers after adjustment for age and sex. While driving, 80 patients with glaucoma (71.4%) declared never using the mobile telephone (except for emergencies), whereas 30 (26.8%) admitted rarely using and 2 (1.8%) sometimes using it. In the control group, 48 participants (68.6%) declared never using the mobile telephone while driving (except for emergencies), whereas 20 (28.6%) admitted rarely using and 2 (2.9%) sometimes using the telephone while driving (*P* = .80). Thirty-one patients with glaucoma (27.7%) vs 26 controls (37.1%) declared that they felt capable of driving while talking on a handheld telephone, and 7 patients with glaucoma (6.3%) vs 10 controls (14.3%) declared that they felt very capable of the task (*P* = .06).

**Table 1.** Demographic and Clinical Characteristics of Survey Respondents and the Subgroup Who Underwent Driving Simulation

Characteristic	Survey^a^			Driving Simulation^a^		
	Control (n = 70)	Glaucoma (n = 112)	*P* Value	Control (n = 28)	Glaucoma (n = 37)	*P* Value
Age, y	68.4 (10.9)	73.6 (9.6)	.001^b^	64.7 (10.0)	69.1 (11.9)	.09^b^
Female, No. (%)	49 (70.0)	56 (50.0)	.01^c^	15 (53.6)	8 (21.6)	.01^c^
African American, No. (%)	12 (17.1)	21 (18.8)	.85^c^	9 (32.1)	10 (27.0)	.78^c^
VA of better eye, logMAR	0.1 (0.2)	0.2 (0.2)	.01^b^	0 (0.1)	−0.1 (0.1)	.60^b^
VA of worse eye, logMAR	−0.1 (0.1)	−0.1 (0.1)	.13^b^	0 (0.1)	0 (0.2)	.58^b^
SAP 24-2 mean deviation of better eye, dB	0.3 (1.2)	−2.6 (4.1)	<.001^b^	0.6 (1.4)	−2.5 (3.7)	<.001^b^
SAP 24-2 mean deviation of worse eye, dB	−0.5 (1.5)	−6.3 (6.2)	<.001^b^	−0.6 (1.7)	−7.0 (6.4)	<.001^b^
Binocular SAP 24-2 mean sensitivity, dB	31.3 (1.5)	28.1 (3.8)	<.001^b^	31.6 (1.7)	28.4 (3.4)	<.001^b^
Reaction time, s						
Mean	NA	NA	NA	0.92	1.14	.002^d^
Median (IQR)	NA	NA	NA	0.76 (0.67-1.14)	1.05 (0.86-1.24)	
Reaction time while using the telephone, s						
Mean	NA	NA	NA	1.55	1.96	.02^d^
Median (IQR)	NA	NA	NA	1.14 (0.98-1.59)	1.86 (1.42-2.29)	
Telephone task, correct answers, %	NA	NA	NA	91.9 (10.2)	91.7 (9.9)	.95^b^

Abbreviations: dB, decibels; IQR, interquartile range; NA, not applicable; SAP, standard automated perimetry; VA, visual acuity.

^a^ Values are presented as mean (SD) unless otherwise noted.

^b^ Wilcoxon rank sum test.

^c^ Fisher exact test.

^d^ Generalized estimating equation.

Thirty-seven participants with glaucoma and 28 controls agreed to perform the driving simulation experiment (Table 1). There were no statistically significant differences in age, race/ethnicity, and visual acuity between groups. There was a lower number of women in the glaucoma group (8 women [21.6%]) compared with the control group (15 women [53.6%]) (*P* = .01). As expected, eyes with glaucoma had significantly worse results on the SAP 24-2 mean deviation test in the better eye, worse eye, and also on the integrated binocular SAP mean sensitivity compared with controls (Table 1).

Participants with glaucoma exhibited significantly longer reaction times while driving compared with controls. This result was observed both without use of a mobile telephone (mean [median; interquartile range (IQR)] reaction time, 1.14 [1.05; 0.86-1.24] seconds vs 0.92 [0.76; 0.67-1.14] second; *P* = .002) and during the mobile telephone task (1.96 [1.86; 1.42-2.29] seconds vs 1.55 [1.14; 0.98-1.59] seconds; *P* = .02). Moreover, the mean (SD) increase of 0.85 (0.60) second in reaction time elicited with mobile telephone use in patients with glaucoma was significantly greater than the increase of 0.68 (0.83) second for controls, a 25% difference (*P* = .03). Correct responses to the questions on the telephone task did not differ between patients with glaucoma and controls (mean [SD] correct responses, 91.7% [9.9%] vs 91.9% [10.2%]; *P* = .95).

Table 2 shows results of univariable models explaining divided-attention reaction times during the driving task with mobile telephone use from both groups, and the Figure shows the association of reaction times during divided attention with binocular mean sensitivity. Each 5-dB decrease in binocular mean sensitivity was associated with an increase of 0.88 second in reaction time (95% CI, 0.65-1.10 seconds; *P* < .001). Older age was also associated with an increase in reaction time of 0.37 second (95% CI, 0.18-0.55 second; *P* < .001) per 10 years. There was no significant association between reaction times and sex or race. Reaction times in the driving task with mobile telephone use were significantly associated with reaction times without mobile telephone use (coefficient, 0.20; 95% CI, 0.06-0.33; *P* = .005).

**Table 2.** Univariable Linear Regression Models for Explaining Divided-Attention Reaction Times in the Driving Task With Mobile Telephone Use

Variable	Coefficient (95% CI)	*P* Value
SAP 24-2 binocular mean sensitivity, per 5-dB decrease	0.88 (0.65 to 1.10)	<.001
Age, per 10-y increase	0.37 (0.18 to 0.55)	<.001
Female	−0.11 (−0.65 to 0.42)	.68
African American	0.52 (−0.04 to 1.08)	.07
Mean reaction time without telephone, s	0.20 (0.06 to 0.33)	.005

Abbreviation: SAP, standard automated perimetry.

**Figure.**
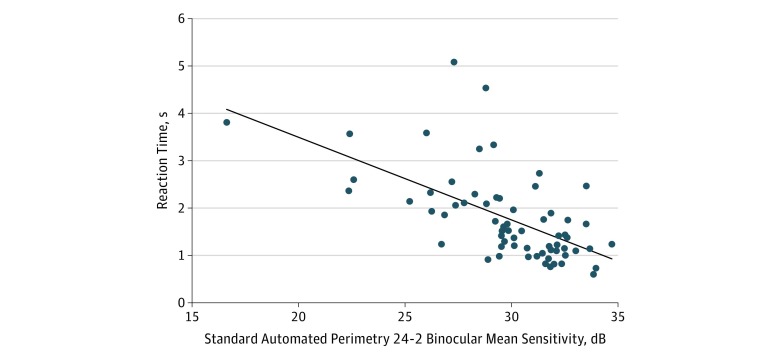
Standard Automated Perimetry Integrated Binocular Mean Sensitivity and Divided-Attention Reaction Times During Driving Task With Mobile Telephone Use

Table 3 shows a multivariable model investigating factors associated with reaction times during driving simulation. The model included disease severity measured by SAP integrated binocular mean sensitivity, a categorical variable indicating whether the task was performed with or without mobile telephone use, as well as a variable indicating an interaction between disease severity and mobile telephone use. The model was adjusted for age and sex. The results showed that the interaction between disease severity and mobile telephone use was significant. In the presence of mobile telephone use, a 5-dB decrease in SAP binocular mean sensitivity was associated with a 0.33-second (95% CI, 0-0.65 second) increase in reaction time compared with no mobile telephone use (*P* = .048).

**Table 3.** Multivariable Model Results for Investigating Factors Associated With Reaction Times During Driving Simulation, Adjusted for Potential Confounding Factors

Variable	Coefficient (95% CI)	*P* Value
SAP 24-2 binocular mean sensitivity, per 5-dB decrease	0.50 (0.30 to 0.73)	<.001
Mobile telephone use, yes	2.68 (0.68 to 4.68)	.01
Interaction between SAP 24-2 binocular mean sensitivity and mobile telephone use	0.33 (0 to 0.65)	.048
Age, per 10-y increase	0.08 (−0.01 to 0.17)	<.001
Female	0.07 (−0.18 to 0.33)	.57

Abbreviation: SAP, standard automated perimetry.

## Discussion

In addition to the significantly greater reaction time required in nondistracted (ie, no mobile telephone use) situations, in this study we showed that, compared with controls, patients with glaucoma were associated with a disproportionately greater increase in reaction time when performing a driving task while using a mobile telephone. These findings indicate that, in the presence of distractors, the driving performance of patients with glaucoma may be impaired to an even greater degree than that of controls. This finding is particularly concerning in terms of safety, especially considering that patients with glaucoma reported a similar frequency of use of a mobile telephone while driving compared with controls, which suggests that they may be largely unaware of their own limitations.

We found that there was a significant association between the severity of glaucomatous damage, assessed by the amount of visual field loss, and the reaction times. Each 5-dB decrease in SAP binocular mean sensitivity was associated with an increase of 0.88 second (*P* < .001) in reaction time during the driving task with mobile telephone use. In a multivariable model, the interaction between mobile telephone use and SAP mean sensitivity was significantly associated with reaction times. This finding indicated that the worse reaction times were seen in patients with glaucoma who exhibited greater visual field loss and when the driving task was performed with the concurrent use of a mobile telephone. This association persisted even after adjustment for age and sex. From the results of this model, an individual with glaucoma exhibiting a moderate loss on SAP with a mean sensitivity of 25 dB would have a reaction time of 1.60 seconds compared with 1.02 seconds for an individual with a relatively preserved field (30 dB). When driving while using a telephone, the participant with moderate visual field loss would present a reaction time of 2.50 seconds and the participant with mild visual field loss would present a reaction time of 1.76 seconds. This disproportionate difference in reaction times between groups is likely to be significant. In real life, drivers travel a certain distance before taking an action to avoid hazards: in the example above, if both individuals drive at 70 mph while talking on a mobile telephone, the driver with greater visual field loss would travel an extra 76 feet (22.8 m) before an action is taken compared with the counterpart who has a relatively preserved visual field.

Our findings were in accordance with previous studies that observed a significantly slower reaction in patients with glaucoma during a divided-attention driving task compared with healthy controls. Reaction time has been shown to be a risk factor in real-world motor vehicle crashes. Gracitelli et al^8^ showed that a reaction time that was 1 SD (0.76 second) above the mean was associated with a 57% increase in the risk for motor vehicle crashes. The important point from the present study is that the decrease in performance while using a mobile telephone was significantly worse for patients with glaucoma compared with controls. Based on the study by Gracitelli et al,^8^ this finding indicated that patients with glaucoma may be at greater risk for motor vehicle crashes, especially when driving during distracted circumstances.

Several mechanisms could explain this disproportionate decrease in driving performance of patients with glaucoma during divided attention. A recent study by Gangeddula et al^9^ showed that the functional field of view among patients with glaucoma results in a disproportionate reduction in performance compared with healthy individuals who have increased cognitive loading, which may help explain the results of our study. Moreover, when drivers are cognitively distracted, their eyes are fixated on a single target and eye dispersion is less.^10^ This means that drivers, while engaged in a telephone conversation, will need to rely on peripheral detection instead of eye movements, which is more challenging to patients with glaucoma because of visual field loss. The other mechanism is that attention can move without eye movements,^24,25^ and it is expected that mechanism is also suppressed during a telephone conversation. Because the visual demand is higher among patients with glaucoma, it is reasonable to suspect that their attention is also more focused because their mobile telephone task performance is not compromised. More focused eye movements and more focused attention during a mobile telephone task as well as compromised peripheral stimulus processing with glaucoma likely explain our results. Attention shifting also may be compromised from damage in the lateral geniculate nucleus,^26,27^ which can lead to degradation of mechanisms associated with release and capture of attention in patients with glaucoma.

Distracted driving due to mobile telephone use is a relevant phenomenon. In the United States, the National Highway Traffic Safety Administration^28^ estimated that, at any point during daytime hours, 481 000 passenger vehicles were driven by people using handheld mobile telephones. Notably, in 2016 there were 3450 distraction-related deaths, which included the use of mobile telephones.^28^ Despite restrictions on the use of mobile telephones while driving in many states, it seems not to be a rare habit. When asked about driving habits and mobile telephone use while driving, patients with glaucoma gave responses that were similar, on average, with controls’. This finding suggests that patients with glaucoma may be unaware of their increased relative difficulty with divided-attention tasks since glaucoma remains relatively asymptomatic until the later stages. However, the results of our study indicate that driving performance may be compromised even before patients are aware, suggesting the need for objective tasks for performance evaluation that are predictive and easy to administer.

To our knowledge, this study is the first to test divided attention in patients with glaucoma in a driving simulator while using a mobile telephone. The high-fidelity simulator enables valid realistic simulations of complex on-road situations in a low-cost and safe way. Although limited physical, perceptual, and behavioral fidelity in simulation can be an issue, the possibility of safely gathering unique measurements for research purposes outweighs these disadvantages. In addition, data from on-road and simulation studies have been compared previously to assess the validity of measures generated in simulators.^29,30^ Wang et al^30^ compared 2 different types of simulators with an on-road test and found that a simulator similar to ours provided a safe and effective way to evaluate driver behavior. The outcomes of mobile telephone use have also been studied previously in a healthy population using simulators, with Woo and Jawkuan^31^ reporting worse performance, as assessed by reaction time, when using a mobile telephone.

### Limitations

The task was limited to handheld telephone use in the simulator. However, the risk of motor vehicle crashes with hands-free devices is not significantly different from that with handheld telephones.^32^ Also, our results may have been influenced by a speed-accuracy trade-off, which refers to the give-and-take relationship between an individual’s responding more quickly and making relatively more errors compared with responding more slowly and make relatively fewer errors. Patients with glaucoma may have chosen to perform more accurately in the task in exchange for being slower. However, the performance in the telephone task was the same for both groups, which indicates similar accuracy. Therefore, it does not seem likely that our results were largely influenced by a speed-accuracy trade-off. Some factors involved in driving performance (eg, contrast sensitivity, location of damage in visual field, and driving history of previous motor vehicle crashes)^33,34^ were not evaluated in this study and should be considered in future studies.

## Conclusions

Our results showed that patients with glaucoma are associated with a significant increase in reaction time while driving and talking on a mobile telephone (vs no mobile telephone) compared with individuals without glaucoma. They also indicated that patients with visual field loss from glaucoma may be unaware of their higher risk while driving, which is worsened by routine distractions such as mobile telephone calls. This higher risk raises the concern for safety and indicates a potential need for clinical evaluation of visual performance under conditions of increased cognitive load, such as when driving while using a mobile telephone.

## Notes

Supplement. **eFigure.** High-Fidelity Driving Simulator
