# Supplementary material for: Mobile Telephone Use and Reaction Time in Drivers With Glaucoma
Source: JAMA Netw Open. 2019 Apr 12;2(4):e192169. doi: 10.1001/jamanetworkopen.2019.2169 (PMC6481446; doi:10.1001/jamanetworkopen.2019.2169)
Supplement: Supplement. — eFigure. High-Fidelity Driving Simulator [file jamanetwopen-2-e192169-s001.pdf]

Supplementary Online Content

Ogata NG, Daga FB, Jammal AA, et al. Mobile telephone use and reaction time in drivers with glaucoma. *JAMA Netw Open*. 2019;2(4):e192169. doi:10.1001/jamanetworkopen.2019.2169

eFigure. High-Fidelity Driving Simulator

This supplementary material has been provided by the authors to give readers additional information about their work.

© 2019 Ogata NG et al. *JAMA Network Open*.

eFigure. High-Fidelity Driving Simulator

High-fidelity driving simulator (Realtime Technologies, Inc., Royal Oak, MI) used in the experiment. Full-size Ford Fusion cab and the visual system screens (A). Diamond-shaped targets (B) were presented peripherally at approximately 20 degrees of visual angle during a simulating driving test. Participants were instructed to press a button on the steering wheel when the stimuli changed randomly either on the left (C) or right hand side. Reaction times were measured to access the ability to divide attention in the driving experiment.

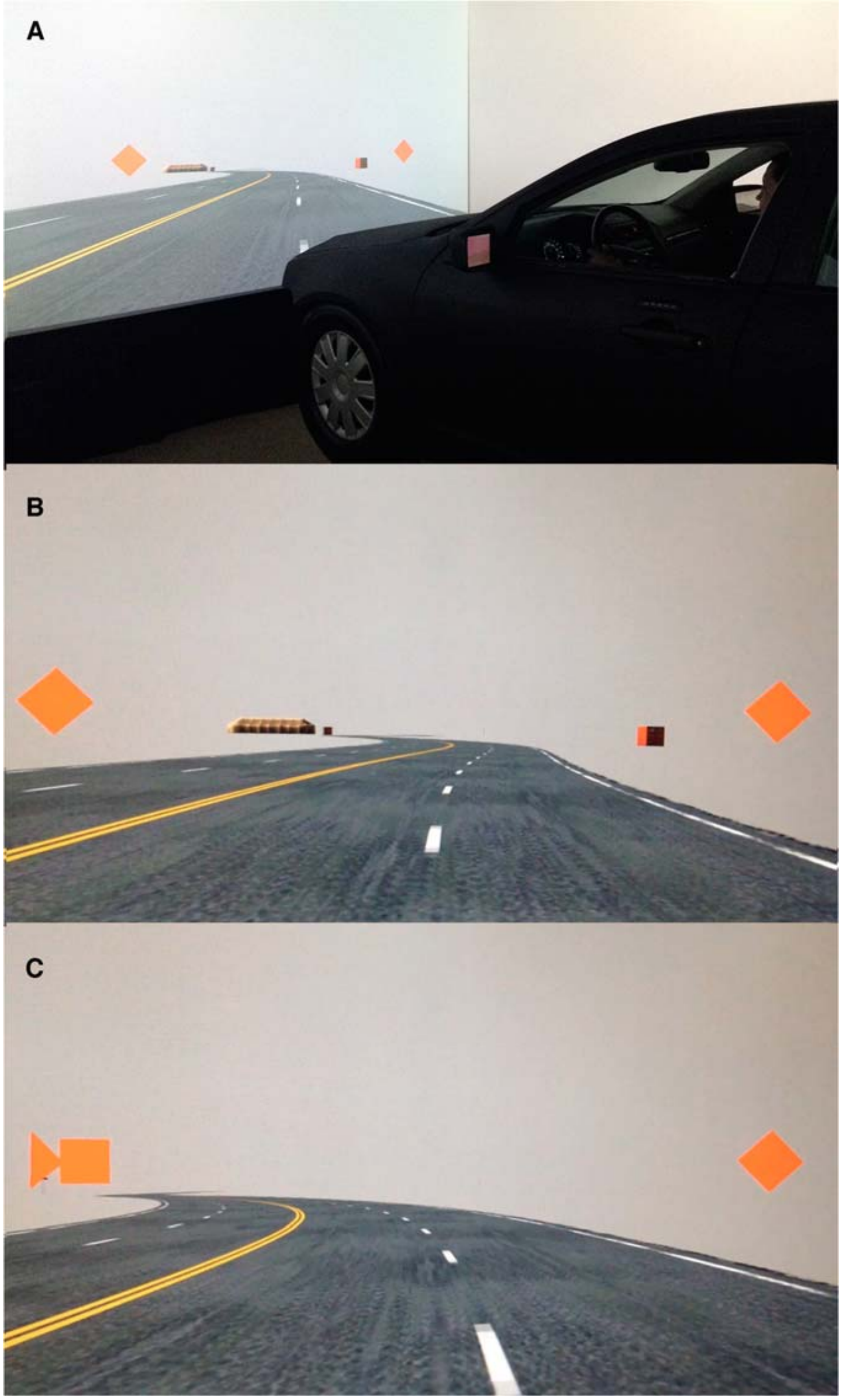

© 2019 Ogata NG et al. *JAMA Network Open*.
